# The effect of the combination of acids and tannin in diet on the performance and selected biochemical, haematological and antioxidant enzyme parameters in grower pigs

**DOI:** 10.1186/1751-0147-52-19

**Published:** 2010-03-06

**Authors:** Marina Štukelj, Zdravko Valenčak, Mladen Krsnik, Alenka Nemec Svete

**Affiliations:** 1 University of Ljubljana, Veterinary Faculty, Institute for health care of pigs, Gerbičeva 60, 1000 Ljubljana, Slovenia; 2 University Medical Centre Ljubljana, Institute of clinical chemistry and biochemistry, Njegoševa 4, 1525 Ljubljana, Slovenia; 3 University of Ljubljana, Veterinary faculty, Clinic for small animal medicine and surgery, Gerbičeva 60, 1000 Ljubljana, Slovenia

## Abstract

**Background:**

The abolition of in-feed antibiotics or chemotherapeutics as growth promoters have stimulated the swine industry to look for alternatives such as organic acids, botanicals, probiotics and tannin. The objective of the present study was to compare the effects of a combination of acids and tannin with diet with organic acids and diet without growth promoters on the growth performance and selected biochemical, haematological and antioxidant enzyme parameters in grower pigs. Tannin is more natural and cheaper but possibly with the same effectiveness as organic acids with regard to growth performance.

**Methods:**

Thirty-six 7 week old grower pigs, divided into three equal groups, were used in a three week feeding trial. Group I was fed basal diet, group II basal diet with added organic acids and group III basal diet with added organic and inorganic acids and tannin. Pigs were weighed before and after feeding and observed daily. Blood was collected before and after the feeding trial for the determination of selected biochemical, haematological and antioxidant enzyme parameters. One-way ANOVA was used to assess any diet related changes of all the parameters. Paired t-test was used to evaluate changes of blood parameters individually in each group of growers before and after feeding.

**Results:**

No clinical health problems related to diet were noted during the three week feeding trial. The average daily gain (ADG) and selected blood parameters were not affected by the addition to basal diet of either acids and tannin or of organic acids alone. Selected blood parameters remained within the reference range before and after the feeding trial, with the exception of total serum proteins that were below the lower value of reference range at both times. The significant changes (paired t-test) observed in individual groups before and after the feeding trial are related to the growth of pigs.

**Conclusion:**

Diet with acids and tannin did not improve the growth performance of grower pigs but had no deleterious effects on selected blood parameters. The possibility of beneficial effects of adding acids and tannin in diets on growth performance over a longer period, however, could not be excluded.

## Background

Recent public concern about the use of numerous compounds in animal diets to enhance performance and health and welfare issues, coupled with changes in regulations on the use of synthetic medicaments, has stimulated interest and research into the use and effects of phytochemicals and plant secondary metabolites in the diet of farm animals [1,2].

Enhancement of growth and feed efficacy are critical in modern pig production [3]. For more than 50 years disease suppression and growth promotion have been achieved by the incorporation of various antibiotics or chemotherapeutics at sub-therapeutic doses into pig feeds [4,5]. In January 2006, the use of antibiotics as growth promoters was prohibited in the European Union, largely due to concerns about bacterial resistance to antibiotics and consumer food safety issues. Consequently, the swine industry has been stimulated to look for alternatives to antibiotics, such as organic acids and their salts, short chain fatty acids, naturaceuticals, botanicals, probiotics, tannin, etc [6-11]. Particular interest is now being paid to the antimicrobial potency of various carboxylic acids and of short chain fatty acids [5].

Organic acids have been used for a number of years with varying success for ameliorating enteric infections [9] and the withdrawal of antibiotics has forced them back into focus. Several studies have reported that inclusion of organic acids and/or their salts into pig feed increases growth performance in all classes of pigs [7,11-15]. Diet acidifiers have been reported to reduce bacterial populations in different segments of the gastrointestinal tract of pigs [7,11,14,16,17]. However, reduced scouring has been observed in only a few studies [7,17]. The multifunctional role of organic acids may lead to improved digestion, absorption and retention of numerous dietary nutrients [7,11].

Tannins are defined as naturally occurring, water-soluble polyphenolic compounds, commonly found in higher herbaceous and woody plants. They belong to a major group of antimicrobial compounds from plants, and can also be toxic to filamentous fungi, yeasts, and bacteria. Tannins have also been reported to inactivate certain viruses [6,18-21]. The presence of tannins in diets for livestock have been reported to have anti-nutritional and toxic effects, including reduced feed intake, growth rate, feed efficiency and net metabolizable energy [2,6,19,22-24]. In addition, they are known to form insoluble complexes with metal ions such as iron, rendering them less available for absorption [6,25,26]. However, because of their antioxidant action in scavenging free radicals, chelating transition metals, inhibiting pro-oxidative enzymes and in lipid peroxidation it is possible that tannins are beneficial [19,27,28].

It is well known that aerobic organisms are constantly exposed to reactive oxygen species (ROS) that are produced mainly as a consequence of aerobic respiration in mitochondria and substrate oxidation. In healthy organisms, their production is counterbalanced by the antioxidant defence system. Serious imbalance leading to ROS excess is known as oxidative stress, which is implicated in several diseases. However, antioxidants within cells, cell membranes and extracellular fluids can be up-regulated and mobilized to neutralize excessive ROS formation [29-31]. The enzymatic and non-enzymatic antioxidant defences include superoxide dismutase (SOD), glutathione peroxidase (GPX), catalase (CAT), ascorbic acid (vitamin C), α-tocopherol (vitamin E), glutathione, β-carotene, and vitamin A. The effects of nutrition and different breeding practices on oxidative stress in pigs have been widely studied, since the ability of pigs to neutralize ROS plays a key role in their welfare and performance [32-36].

Despite certain negative aspects, therefore, tannin can have positive effects on growth performance. Performance and different blood constituents reflect the physiological responsiveness of the animals to their internal and external environments, which include feed and feeding [6,32,37-41]. It is known that feed constitutes the major part of the production costs of pork [3]. The combination of acids and tannin decreases the amount of added acids in basal feed and thus lowers the cost of feed. Tannin is cheaper and completely natural. Therefore, the objective of the present study was to determine the effects of the combination of acids and tannin added into basal diet compared with diet with organic acids and basal diet (i.e. diet without growth promoters) on growth performance and selected biochemical, haematological, and antioxidant enzyme parameters in grower pigs. The effects of a combination of acid and tannin in diets on pigs' growth performance and blood parameters have not yet been studied.

## Methods

### Pigs, housing and diets

Thirty-six 7 week old grower pigs (both sexes) of 15.5 ± 1.86 kg (mean ± SD) were used. They were housed in pens (12 per pen) size 1.5 × 3 m with concrete floors, in facilities at the Institute for health care of pigs, Veterinary Faculty, Ljubljana Slovenia. The temperature was between 16°C to 19°C. Pens were cleaned twice daily, when pigs were also fed. Feed was available ad libitum. Water access was ad libitum on water nipples. The pigs were reared according to the Council directive for minimum standards for the protection of pigs (91/630/EEC).

The pigs were randomized into three groups fed different diets for three weeks. Group I (7 females and 5 castrates) were fed basal diet (Table 1), which was commercial feed for growers (Jata Emona; Ljubljana, Slovenia). Group II (8 females and 4 castrates) were fed the same basal diet, with added 0.3% commercial acidificant FraAcidDry (Perstorp Franklin; Waspik, The Netherlands), which contains lactic acid, citric acid, formic acid, fumaric acid and ammonium formate. Group III (8 females and 4 castrates) were fed the same basal diet with added 0.3% additive containing 0.15% tannin extracted from chestnut tree (Tanin Sevnica; Sevnica, Slovenia) and overall 0.15% of 4 acids (lactic acid, citric acid, orthophosphoric acid and L and R malic acid).

**Table 1:** Percentage composition and chemical content (g, mg and IU per kg of feed) of basal diet

	Group I (basal diet)
crude protein (%)	15

crude fat (%)	3.8

crude fibre (%)	4

ash (%)	5.2

lysine (%)	0.8%

vitamin A (IU)	6000

vitamin D3 (IU)	1000

Vitamin E (mg)	40

vitamin K3 (mg)	1

Vitamin B1 (mg)	1

Vitamin B2 (mg)	3

Nicotine acid (mg)	2

Calcium-D-panthotenat (mg)	15

Vitamin B6 (mg)	2.5

Vitamin B12 (mg)	40

folic acid (mg)	0.5

biotin (mg)	0,1

Fe (mg)	80

Cu (mg)	50

Mn (mg)	35

Zn (mg)	70

Co (mg)	0.4

J (mg)	1

Se (g)	0.35

Lignosulphonate (g)	7.5

The pigs were observed throughout the feeding trial and health status was recorded daily. The incidence of diarrhoea and consistency of faeces were recorded daily in order to detect changes in gastrointestinal function that could be related to the diet used. The pigs were weighed at the beginning and end of the three week feeding trial. Morbidity and mortality were recording in all three groups during the trial and growth performance parameter (average daily gain (ADG)) calculated at the end.

All procedures were approved by Ministry of Agriculture, Forestry and Food, Veterinary Administration of the Republic of Slovenia; license No 323-02-663/2005/2.

### Collection of blood samples

Blood was collected twice, at the beginning and end of the feeding trial. Blood was collected from the vena cava cranialis, for determination of biochemical profile and haematological and antioxidant enzyme parameters.

Blood samples for the determination of biochemical profiles were collected into serum separator tubes (Vacuette; Greiner Bio-one, Kremsmunster, Austria) and stood for 15 minutes to clot prior to centrifugation at 1300 g at 4°C for 10 minutes. Serum samples were stored at -20°C until analysed.

Venous blood samples for the determination of complete blood count (CBC) and white cell differential count (WCDC) were collected into tubes with K_3_EDTA anticoagulant (Vacuette; Greiner Bio-One, Kremsmunster, Austria).

Blood samples for determining antioxidant enzyme parameters in whole blood lysates were collected into tubes containing anticoagulant lithium heparin (Vacuette; Greiner Bio-One, Kremsmunster, Austria) and immediately stored at -80°C until analysed.

### Biochemical analyses

Biochemical profiles, which included determination of copper (Cu), iron (Fe) and total serum protein concentrations (protein), were determined using an automated biochemistry analyser Cobas Mira (Hoffman La Roche Ltd, Basel, Switzerland).

### Haematological analyses

CBC was determined immediately after collection with an automated haematological analyser (ABC Vet, Horiba ABX, Montpellier, France). WCDC was determined manually on the same day as CBC. CBC and WCDC included: red blood cells (RBC), haemoglobin (Hgb), mean corpuscular volume (MCV), haematocrit (Ht), white blood cells (WBC), platelets (Plt), neutrophils (Neut), eosinophils (Eos), basophils (Baso), lymphocytes (Lymph), band neutrophils (BN) and monocytes (Mono).

### Measurement of GPX activity

Activity of GPX was measured using the commercial Ransel kit (Randox Laboratories, Crumlin, UK) with an automated biochemical analyser Hitachi 917 (Hitachi, Japan). According to the method GPX activity is determined indirectly by measuring the rate of formation of oxidized glutathione (GSSG). GPX catalyzes the reaction of GSH with synthetic cummene hydroperoxide to GSSG. In the presence of NADPH and glutathione reductase GSSG is transformed to glutathione, and NADPH is oxidized to NADP. The rate of oxidation of NADPH was measured spectrophotometrically as reduced absorbance at 340 nm and is proportional to the activity of GPX in the specimen. Activity of GPX was expressed as Units/g of haemoglobin (U/g Hgb).

### Measurement of SOD activity

SOD activity was determined spectrophotometrically (550 nm) with an automatic biochemical analyser Hitachi 917 (Hitachi, Japan), using commercially available Ransod kit (Randox Laboratories, Crumlin, UK). According to the method the superoxide radicals were generated by the xanthine and xanthine oxidase reaction. The amount of superoxide radical produced was determined by 2-(4-iodophenyl)-3-(4-nitrophenol)-5-phenyltetrazolium chloride (INT) as an indicator, which reacts with a superoxide radical to form formazan dye. The SOD activity was determined by the grade of inhibition of the described reaction. The standard calibration curve of percentage of inhibition by standard solutions and log concentrations (U/ml) was used to determine SOD activity in our specimens. Activity was expressed as U/g Hgb.

### Statistical analysis

Data were analysed using the SPSS computer program (SPSS 15.0 for Windows, Chicago, Illinois, USA). Results are expressed as mean ± standard deviation (mean ± SD). At the age of 7 weeks the body weight and values of selected blood parameters (haematological, biochemical and antioxidant) were compared between the three groups of growers, using one-way ANOVA. The same statistical method was used to determine the effect of different diets on the growth performance parameter (ADG) and on selected blood parameters (haematological, biochemical and antioxidant) of grower pigs after the trial. Paired t-test was used to compare selected blood parameters before and after feeding, individually for each group of growers, to evaluate changes of selected parameters in individual groups. The minimum level of significance was defined at p < 0.05.

## Results

At the beginning of the feeding trial one pig died during blood sampling in Group I. No clinical health problems related to diet were noted during the three week feeding trial. The incidence of diarrhoea and consistency of faeces were recorded daily in order to detect changes in gastrointestinal function that could be related to the diet used. Transitory soft faeces were observed five times in group I and eleven times in groups II and III.

Growth data are summarized in Table 2. At the beginning of the trial one-way ANOVA showed no statistically significant differences in body weight (p = 0.990) between the groups of growers. No statistically significant difference in ADG (p = 0.692) was observed between groups at the end of the trial. The highest ADG (numerically) was recorded in Group I (basal diet) - 1.053 ± 0.217 kg versus 0.987 ± 0.282 kg (Group II) and 0.969 ± 0.230 kg (Group III).

**Table 2:** Body weight (BW; mean ± SD) and average daily gain (ADG; mean ± SD) in three groups of 7 week old growers before (initial BW) and after (final BW) three week feeding trial

	Initial BW (kg)	Final BW (kg)	ADG (kg)
group I	15.62 ± 1.61	37.64 ± 5.25	1.053 ± 0.217
group II	15.56 ± 1.88	36.29 ± 6.92	0.987 ± 0.282
group III	15.51 ± 2.19	35.85 ± 5.55	0.969 ± 0.230
p value (one-way ANOVA)	0.990	0.761	0.692

Data analysis (one-way ANOVA) showed no statistically significant differences in any of selected blood parameters between the three groups of growers, neither at the beginning nor the end of the trial. However, most of the selected blood parameters, individually for each group, differed significantly between the beginning and end of the feeding trial (Tables 3, 4, 5 and 6).

**Table 3:** Biochemical parameters (mean ± SD) in three groups of 7 week old growers before (Initial values), and after (Final values) feeding trial

	Initial values	Final values	**Reference range **[42]
**protein **(g/L)			79 - 89
group I	52.2 ± 2.6*	66.8 ± 3.2	
group II	53.7 ± 4.2*	64.3 ± 6.0	
group III	51.9 ± 4.4*	63.1 ± 3.5	

p value (one-way ANOVA)	0.449	0.139	

**Fe **(μmol/L)			16.3 - 35.6
group I	17.60 ± 5.85*	23.0 ± 8.10	
group II	19.73 ± 6.78	21.96 ± 5.56	
group III	16.97 ± 7.31	19.62 ± 6.30	

p value (one-way ANOVA)	0.573	0.462	

**Cu **(μmol/L)			20.9 - 43.8
group I	26.17 ± 5.29*	31.25 ± 2.42	
group II	29.75 ± 3.36	30.76 ± 2.93	
group III	27.25 ± 7.10	30.97 ± 3.05	

p value (one-way ANOVA)	0.272	0.916	

* p < 0.05, paired t-test used for the comparison between initial and final values in individual groups of growers

**Table 4:** Complete blood count parameters (mean ± SD) in three groups of 7 week old growers before (Initial values) and after (Final values) feeding trial

	Initial values	Final values	**Reference range **[44]
**WBC (10^9^/L)**			18.9 - 26.9
group I	30.08 ± 11.46*	18.96 ± 2.88	
group II	23.72 ± 4.33*	19.26 ± 4.77	
group III	27.78 ± 4.94*	19.86 ± 3.82	
p value (one-way ANOVA)	0.134	0.855	
**RBC (10^12^/L)**			5.0 - 8.0
group I	5.84 ± 0.44*	6.77 ± 0.42	
group II	5.87 ± 0.18*	6.60 ± 0.57	
group III	5.92 ± 0.31*	6.59 ± 0.49	
p value (one-way ANOVA)	0.810	0.628	
**Hgb (g/L)**			100 - 160
group I	99.7 ± 0.8*	116.7 ± 0.7	
group II	100.2 ± 0.5*	113.6 ± 0.9	
group III	99.6 ± 0.7*	109.8 ± 0.6	
p value (one-way ANOVA)	0.973	0.155	
**MCV (fL)**			50 - 68
group I	58.3 ± 2.9*	55.6 ± 2.9	
group II	58.4 ± 1.6*	55.2 ± 1.3	
group III	57.6 ± 2.8*	54.1 ± 4.3	
p value (one-way ANOVA)	0.678	0.512	
**Ht (L/L)**			0.32 - 0.50
group I	0.342 ± 0.027*	0.376 ± 0.024	
group II	0.341 ± 0.016*	0.364 ± 0.031	
group III	0.341 ± 0.025	0.355 ± 0.018	
p value (one-way ANOVA)	1.000	0.643	
**Plt (10^9^/L)**			325 - 715
group I	511.6 ± 115.4*	351.9 ± 76.9	
group II	502.0 ± 155.3*	331.1 ± 40.5	
group III	514.5 ± 123.3*	367.1 ± 129.0	
p value (one-way ANOVA)	0.972	0.643	

* p < 0.05; paired t-test used for the comparison between initial and final values in individual groups of growers

**Table 5:** White cell differential count parameters (mean ± SD) in three groups of 7 week old growers before (Initial values) and after (Final values) feeding trial

	Initial values	Final values	**Reference range **[44]
**Neut (%)**			28 - 47
group I	61.6 ± 16.0*	34.4 ± 11.8	
group II	56.4 ± 10.1*	34.9 ± 12.6	
group III	61.1 ± 7.5*	38.0 ± 14.3	
p value (one-way ANOVA)	0.503	0.769	
**Lymph (%)**			39 - 62
group I	36.6 ± 16.0*	57.4 ± 12.0	
group II	40.1 ± 10.4*	59.1 ± 11.6	
group III	36.3 ± 7.8*	55.5 ± 13.6	
p value (one-way ANOVA)	0.692	0.790	
**Mono (%)**			1.0 - 5.0
group I	0.75 ± 0.97*	2.09 ± 1.70	
group II	1.25 ± 1.55	1.82 ± 1.60	
group III	1.08 ± 1.51	1.42 ± 1.38	
p value (one-way ANOVA)	0.662	0.585	
**Eos (%)**			0.5 - 2.5
group I	0.36 ± 0.67*	5.55 ± 5.72	
group II	0.36 ± 0.51*	3.82 ± 2.52	
group III	0.25 ± 0.62*	4.25 ± 2.26	
p value (one-way ANOVA)	0.924	0.545	
**Baso (%)**			0.0 - 0.5
group I	0.00 ± 0.00	0.09 ± 0.30	
group II	0.00 ± 0.00	0.00 ± 0.00	
group III	0.17 ± 0.58	0.25 ± 0.62	
p value (one-way ANOVA)	0.379	0.394	
**BN (%)**			1.0 - 2.0
group I	0.83 ± 1.19	0.55 ± 1.29	
group II	1.92 ± 1.44*	0.18 ± 0.41	
group III	1.08 ± 1.38	0.50 ± 1.17	
p value (one-way ANOVA)	0.134	0.670	

* p < 0.05; paired t-test used for the comparison between initial and final values in individual groups of growers

**Table 6:** SOD and GPX values (mean ± SD) in three groups of 7 week old growers before (Initial values) and after feeding trial (Final values)

	Initial values	Final values	Initial values	Final values
	
	SOD (U/g Hgb)	SOD (U/g Hgb)	GPX (U/g Hgb)	GPX (U/g Hgb)
group I	1499.3 ± 226.9	1473.9 ± 145.9	198.9 ± 47.3	225.9 ± 48.5
group II	1357.7 ± 175.3	1342.3 ± 205.0	192.6 ± 32.8	210.2 ± 46.0
group III	1343.0 ± 79.8*	1442.9 ± 84.6	184.0 ± 35.1	227.4 ± 58.5
p value (one-way ANOVA)	0.138	0.128	0.682	0.704

*p < 0.05, paired t-test used for the comparison between initial and final values in individual groups of growers

Among the biochemical parameters (Table 3), the level of protein, an indicator of adequacy of protein in terms of quality and quantity in the diet [42], at the beginning of the trial was below the lower value of the reference range [43] in all three groups of growers. After three weeks feeding it had increased significantly in all three groups, though the values remained below the lower value of reference range. Fe and Cu concentrations were within the reference ranges [43] in all three groups of growers before and after the feeding trial, but increased significantly (paired t-test) in the group of growers fed the basal diet only (Group I).

No diet related changes of haematological parameters (Tables 4 and 5) were found (one-way ANOVA) after the three weeks feeding trial. Initial and final values of most of the haematological parameters were within their reference ranges [44]. Comparison of the results before and after the trial, individually for each group of growers (paired t-test), showed statistically significant increases of RBC, Hgb, Lymph and Eos, and decreases of MCV, Plt, WBC, Neut, in all three groups of growers. At the end of the trial significant increases were observed for Ht in groups I and II, Lymph in groups I and III and Mono in group I. BN was significantly lower only in group II.

GPX and SOD activities showed no diet related changes after the three weeks feeding trial (Table 6) and were in keeping with published values [45,46]. When comparing SOD and GPX activities before and after the feeding trial, individually for each group of growers, the results of statistical analysis (paired t-test) showed significant difference in SOD activity only in the group that was fed basal diet with added acids and tannin (Group III). GPX activity increased after the three weeks feeding trial in all groups, however, the difference was not significant.

## Discussion

Feed constitutes the major part of the production costs of pork [3]. In the present study, the combination of acids and tannin allows the amount of expensive organic acids to be decreased. Tannin is completely natural in comparison with acids, but possibly with the same effectiveness as organic acids with regard to growth performance of grower pigs. However, the results of the study showed that the addition of acids and tannin into basal diet did not improve the growth performance of grower pigs but had no deleterious effects on biochemical, haematological and antioxidant parameters.

In the present study, growth performance parameter, ADG, did not differ significantly between the diets used, which clearly indicates that neither the combination of acids and tannin nor organic acids added into basal diet improved growth performance. The result is in contrast to previous studies, which reported improved ADG in response to inclusion of organic acids and their salts into diet during grower period [11,13,14,17,47]. In our study ADG numerically was the highest in the group fed basal diet and the lowest in the group fed basal diet with added acids and tannin. Though the difference between diets was not statistically significant, the lowest ADG could be ascribed to tannin properties. It has been reported that tannins present in diets for livestock reduce feed intake, growth rate, feed efficiency and net metabolizable energy [2,6,19,23].

The lack of a response on ADG in the group of growers fed diet with added organic acids in our study in comparison with studies that reported improvement of growth performance could be related to differences in the combination and dose of the acids, composition of basal diet, age of animals and existing levels of performance. Each acid has unique properties, including its pK_a_, and therefore the results cannot be generalized from one acid and their salts to another or to the combination of organic acids. In addition, even different salts of formate affect animal response. It has been reported that K-diformate was more effective in improving ADG and feed conversion efficiency than Na-Ca-formate [14].

On the other hand, the lack of a response on ADG in our study might also be ascribed to a relatively short feeding period, from age of 7 to 10 weeks. In some experiments, performance response to inclusion of organic acids and their salts increased with time on feed. A trend for improved efficiency of gain was observed in pigs fed a diet containing formic acid [48] or formic acid-ammonium formate [49] when the entire grower-finisher period was included in the analyses rather than the grower period only. In addition, the lack of a response on ADG to inclusion of organic acids and their salts has also been reported in grower pigs [15,16,50].

Different blood constituents, as well as performance, reflect the physiological responsiveness of the animals to its internal and external environment, which include feed and feeding [6,32,37-41]. In the present study, selected biochemical (protein, Fe and Cu), haematological (CBC and WCDC) and antioxidant enzyme (SOD, GPX) parameters remained within the published values and did not differ statistically significantly between groups of growers before and after the feeding trial. The results clearly indicate that the diet with acids and tannin had no deleterious effects on selected blood parameters and that there were no diet related changes of selected blood parameters.

Among biochemical parameters, protein concentration was under the lower value of reference range [39,43] in all three groups before feeding, which may be due to influence of age or indicate improper feeding/diet of growers in the period before the beginning of the experiment [42]. On the other hand, similar result has already been reported for grower pigs [40]. Total serum protein concentration is an indicator of adequacy of protein in terms of quality and quantity in the diet. It was confirmed that blood proteins depend on the quality of dietary proteins [39,40]. Therefore, it is difficult to compare results, as total serum protein may vary greatly due to different feeding practices and different pig genotypes even at the same age of pigs. After the three weeks feeding trial, irrespectively of the diet used, protein increased significantly, which is most probably due to advanced age [42]. However, the concentrations still remained below the lower value of reference range. In all animals, there is a general increase in total serum protein, a decrease in albumin, and an increase in globulins with advancing age [42].

Fe and Cu concentrations were within the reference ranges [43] in all three groups of growers before and after feeding trial, but increased significantly (paired t-test) in the group of growers fed the basal diet only (Group I).

Although one-way ANOVA did not show diet related changes of any of blood parameters, the lowest values of protein, Fe and Hgb determined in the group of growers fed the diet with added acids and tannin could be ascribed to effects of tannin. In diets for humans and nonruminant animal species, tannins can reduce the digestibility of proteins, increase the excretion of proteins and essential amino-acids, may lower the activity of digestive enzymes, may cause damage to mucosa of the digestive tract or exert systemic toxic effects, and form insoluble complexes with metal ions such as iron, rendering them less available for absorption [2,6,19,22,23,25,26].

At age of 7 weeks, before the feeding trial, some haematological parameters deviated from reference ranges, which was not clinically important [44]. High WBC in Group I and III and Neut values in all three groups and low Lymph in the group I and III could be ascribed to inflammation, which can be the result of infection after a decrease of specific maternal antibodies at this age.

As mentioned above, no diet related changes of haematological parameters were detected. On the other hand, the three weeks feeding trial resulted in statistically significant changes (p < 0.05; paired t-test) of all haematological parameters in individual groups of growers. Despite significant changes, all haematological parameters remained within the reference ranges in all three groups [44]. An increase of RBC, Hgb, Lymph and Eos final values and a decrease of Plt, Neut and BN final values in all three groups may be a consequence of growth of pigs [44]. Changes of Lymph, Plt and Neut values may not be attributed only to growing of pigs but also to presence of mycotoxins in all three diets [44].

Pigs might experience oxidative stress due to the effects of nutrition and different breeding practices. Despite the unfavourable effects of tannins, they could be beneficial due to their antioxidant action, like free radical scavenging activity, chelation of transition metals, inhibition of pro-oxidative enzymes and lipid peroxidation [19,27,28]. Values of SOD and GPX were in agreement with published data for SOD and GPX in pigs [45,46] at initial and final measurements. SOD and GPX are important antioxidant defences, as these enzymes are involved in the clearance of superoxide and hydrogen peroxide [30]. SOD activity with respect to haemoglobin is remarkably constant among a range of vertebrates animals in comparison with GPX and catalase [45]. Our study demonstrated no diet related changes in SOD and GPX activity. There was, however, a statistically significant difference (paired t-test) between initial and final SOD activity in the group fed the diet with added acids and tannin. We may speculate, that this result reflects elevated oxidative stress, thus compensatory mechanism probably resulted from increased superoxide radical generation [30,31].

Though not significantly, values of GPX were higher after feeding in all three groups, which most likely may be a result of increased values of RBC due to growth as GPX measured is predominantly present in erythrocytes.

## Conclusion

Values of ADG and selected blood parameters did not differ significantly between three groups of growers after the three weeks feeding, clearly indicating no diet effects. However, the inclusion of organic acids and the combination of acids and tannin had no deleterious effects on haematological, biochemical and antioxidant enzyme parameters in grower pigs. Selected blood parameters remained within the reference range.

The possibility of beneficial effects of acids and tannin given in diets over a longer period could not be excluded.

## Competing interests

The authors declare that they have no competing interests.

## Authors' contributions

MŠ and ZV have drafted the manuscript and participated in the organisation of experimental design and carried out blood sample collection and testing. MK has performed all the antioxidants status analyses and he has helped to draft the manuscript. ANS has contributed to the organisation of experimental design and to writing the manuscript. She has also made the statistics. The final manuscript was approved by all authors.
